# Comparison study of the response with botulinum toxin muscle injection in the ICR mice from three different sources

**DOI:** 10.1186/s42826-019-0010-4

**Published:** 2019-07-26

**Authors:** Min-Soo Seo, Young-In Kim, Kyung-Ku Kang, Se-Kyung Oh, Soo-Eun Sung, Young-Suk Jung, Joon Yong Cho, HyunKeun Song, Dae Youn Hwang, Sang-Joon Park, Kil Soo Kim

**Affiliations:** 1 Laboratory Animal Center, Daegu-Gyeongbuk Medical Innovation Foundation, Daegu, Korea; 2 KPC Coporation, Gwangju, 12773 Korea; 3 Department of Pharmacy, College of Pharmacy, Pusan National University, Busan, Korea; 4 Department of Health and Exercise Science, Korea National Sport University, 88-15 Oryung-dong, Songpa-gu, Seoul, Korea; 5 Central Research Institute, Kine sciences, F1, Milovany, 28, Inchon-ro, Seongbuk-gu, Seoul, Korea; 6 Department of Biomaterials Science, College of Natural Resources & Life Science/Life and Industry Convergence Research Institute, Pusan National University, Miryang, Korea; 7 Laboratory of Histology, College of Veterinary Medicine, Kyungpook National University, Daegu, Korea; 8 College of Veterinary Medicine, Kyungpook National University, Daegu, Korea

**Keywords:** Korl:ICR, Botulinum toxin, Muscle, ICR mouse

## Abstract

Botulinum-toxin A (BoNT/A) is a widely used not only for cosmetics but also for various experimental purposes including muscle-related research. In this study, we applied BoNT/A to mouse muscle of three different sources to compare and evaluate the biological and pathological response. The three different mouse sources consist of Korl:ICR (Korea FDA source), A:ICR (USA source) and B:ICR (Japan source) which were purchased from each different vendors. To compare the responses of ICR mice with BoNT/A muscle injection, we examined the body weight, hematological and serum biochemistry analysis. Also, we evaluated the muscle change by histopathological analysis and gene expression patterns of muscle-related target by qPCR. The body weight gain was decreased in the BoNT/A-treated group compared with the control group. In clinical pathologic analysis and gene expression patterns, the data showed that the responses in the BoNT/A-treated group were similar compared with the control group. Decreased muscle fiber was observed in BoNT/A-treated group compared with control group, while Korl:ICR showed a little low response with the other mouse sources. In conclusion, our results suggest that three different sources ICR mice (Korl:ICR, A:ICR and B:ICR) have a similar biological and pathological responses in BoNT/A muscle injection.

## Introduction

Botulinum toxin is known to be one of the most potent toxins in nature [1, 2]. Due to its potent toxicity, it has been classified as a highest priority bioterrorism agent by the US Centers for Disease Control and Prevention [3]. Human casualties due to botulinum toxin are reported every year. On the other hand, botulinum toxin is used to treat several diseases including strabismus, urinary incontinence, anal fissure, cervical dystonia, migraine headaches [4–8], and a variety of other diseases [9]. Botulinum toxin is also one of the most commonly used materials in the field of plastic surgery, constituting a billion-dollar industry [10].

Botulinum toxin is a type of protein released by a bacterium known as *Clostridium botulinum*; it acts by reversibly binding to nerves synapses, inhibiting the release of acetylcholine at nerve junctions [11]. This blocks muscle contractions, resulting in secondary muscle relaxation effects, this property has been exploited in the treatment of muscle tension-related diseases [9].

Botulinum toxin is classified into eight subtypes (A, B, C_1_, C_2_, D, E, F and G) based on immunological characteristics [12]. Type A is widely used because it is the most effective [12]. Previous studies have reported the purification [13] of botulinum-toxin A (BoNT/A) and its amino acid composition [14].

Outbred mice and rat stocks are widely used in research and industry. The ICR mice, also known as Swiss mice, were originated from albino mice in Switzerland, and were established at Institute for Cancer Research [15]. Rodents, including ICR mice, have been commonly used as animal models over a prolonged period. BoNT/A potency tests are done using rodents, especially ICR mice [16–18], and include toxicity, nerve, muscle and biological response tests [19–22]. Among laboratory animals, ICR mice are the most commonly used models.

In the present study, we compared the effects of BoNT/A muscle injection in ICR mice obtained from three different sources (Korl:ICR, A:ICR and B:ICR). Further, we evaluated the response and phenotype in Korl:ICR mice, established by the Korea FDA. Our results are the first evidence showing that Korl: ICR, A;ICR, and B:ICR mice have largely similar responses to BoNT/A muscle injection.

## Methods/experimental

### Animals

Male ICR mice (six-week-old) were obtained from three different sources. The ICR mice were purchased from different vendors located in Korea (Korl:ICR), the United States (A:ICR) and Japan (B:ICR). The animal experimental protocols were reviewed and approved by the Institutional Animal Care and Use Committee of KPC Co. Ltd. Kyunggido, korea (KPC-IACUC; approval No. P181112) and were in accordance with their guidelines. All mice were provided with ad libitum access to a standard irradiated chow diet (Purina, Seoul. Korea) and sterilized water. During the study period, the mice were maintained in specific pathogen-free (SPF) conditions under a strict light cycle (light on at 7:00 and off at 19:00, 12-h dark-light cycle), 23 ± 2 °C, and (50 ± 10%) relative humidity at the laboratory animal facility of KPC.

### Experimental design

The three types of mice (Korl:ICR, A:ICR, and B:ICR) were each divided into two groups (negative control group and botulinum toxin-induced group; *n* = 10 in each group). Before the experiment, all mice were acclimatized for 7 days. Botulinum-toxin A (BoNT/A, Botox, 100 U, Allergan, Irvine, CA) was injected into the botulinum toxin-induced group mice and saline into the negative control group mice. The left hind limbs were shaved and a single injection of 1.0 U/kg BoNT/A into the gastrocnemius muscle. Body weight was measured daily for 3 days using an electrical balance (FX-2000i, AND, Japan). After the experiment, all mice were anesthetized using 2% isoflurane (Virbac, UK) in a chamber. Blood samples were collected from the abdominal aorta and stored into a BD Microtainer Blood Collection Tube (BD Life Sciences, Franklin Lakes, NJ, USA) for hematology and serum biochemistry analysis. The muscle tissues were extracted for histopathology analysis.

### Serum biochemistry analysis

For serum biochemistry analysis, the collected blood samples were centrifuged at 3000 g for 15~20 min for serum isolation. Levels of a total of 20 markers were tested including aspartate aminotransferase (AST), alanine aminotransferase (ALT), alkaline phosphatase (ALP), blood urea nitrogen (BUN), creatinine (CRE), glucose (GLU), total cholesterol (CHO), total protein (TP), high density lipoprotein (HDL), low density lipoprotein (LDL), albumin (ALB), total bilirubin (TBIL), triglyceride (TG), calcium (Ca), inorganic phosphorus (IP), sodium (Na), potassium (K), chloride (Cl), phospholipid (PL) and high-sensitive C-reactive protein (hsCRP). The analysis was measured by automated biochemistry analyzer (TBA-120FR, Toshiba, Japan).

### Quantitative real-time polymerase chain reaction

Total RNA was extracted from mouse muscle samples using the RNeasy minikit (Qiagen, Germany) following the manufacturer’s standard protocol. The concentration of extracted RNA was evaluated using a Nanodrop 2000 (ThermoScientific, USA). cDNA was prepared from total RNA via reverse transcription using Superscript II reverse transcriptase (Invitrogen, USA) and oligo dT primers (Invitrogen, USA). Quantitative Real-time PCR (qRT-PCR) was performed by mixing cDNA with primers and LightCycler® 480 SYBR Green I Master (Roche Diagnostics, Germany). qRT-PCR was performed using an LightCycler 480 II with supplied software (Roche applied science, Germany) according to the manufacturer’s instructions. The RNA expression levels were compared after normalization to endogenous glyceraldehyde-3-phosphate dehydrogenase (GAPDH) RNA levels. The primer sequences used are listed in Table 1.

**Table 1:** List of PCR primers

Genes	Forward primer (5′-3′)	Reverse primer (5′-3′)
*Gapdh*	TGGAGAAACCTGCCAAGTATG	GGAGACAACCTGGTCCTCAG
*Col1a1*	TGGTGACAAGGGTGAGACAG	CAGGAGAACCAGGAGAACCA
*Il-6*	CCGGAGAGGAGACTTCACAG	TGCCATTGCACAACTCTTTT
*Tgf-b1*	AGTGGCTGAACCAAGGAGAC	GCTGATCCCGTTGATTTCC
*Mmp-2*	ACACTGGGACCTGTCACTCC	CCAAATAAACCGGTCCTTGA
*MHCIIA*	GCAAACACGAGAGACGAGTG	ATTGTTCCTCAGCCTCCTCA
*MHCIIB*	GGAATGCTGAAGGACACACA	TCTGTCTGTTCCAGGGATGC
*MHCIIX*	GGAGATAAAGGCCAAGAGTGC	AGCCTTGGCTTCCTGTTCTT

### Hematological analysis

The collected whole blood samples were used for hematological analysis. A total of nine parameters were assessed including red blood cell (RBC), hemoglobin (HGB), hematocrit (HCT), mean corpuscular volume (MCV), mean corpuscular hemoglobin (MCH), mean corpuscular hemoglobin concentration (MCHC), platelet (PLT), reticulocyte (RET) and white blood cell (WBC). The analysis was measured by automated hematological analyzer (ADVIA2120i, Siemens, Germany).

### Histopathological analysis

Following the experiments, the muscles were harvested from the mice and fixed with 10% neutral buffered formalin solution for 1 week. The fixed tissues were embedded in a paraffin block and sectioned into 4-μm-thick section. The sections were mounted on slides and stained with hematoxylin and eosin (H&E) solution. The stained slides were observed under a light microscope (TE2000, Nikon, Japan). For histopathology image analysis, the image analyzer software Image J (NIH) was used.

### Statistical analysis

The data were analyzed with Student’s t-tests using Excel (Microsoft, USA) and were represented as mean ± standard error in graphical plots. Statistically significant differences are indicated using asterisks (***p < 0.001, **p < 0.01, *p < 0.05).

## Results

### Body weight

**Fig. 1:**
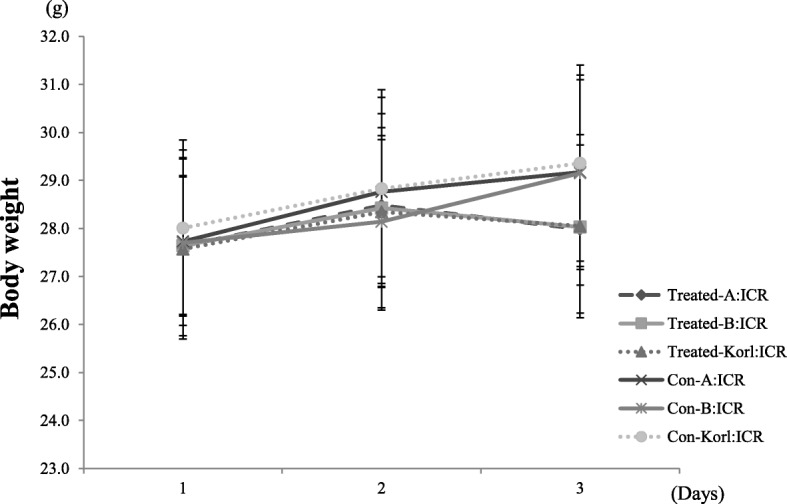
Body weight changes in BoNT/A muscle injection with ICR mice from three different sources for 3 days. The data represent the mean ± SD (*n* = 10/group)

To compare changes in body weight during the experiment period, we measured the body weight for 3 days (Fig. 1). At the beginning of the experiment, the mice had similar body weights. Normal ICR mice (negative control groups) showed continuous and constant weight gain for 3 days during the experiment. On day 3, mice in the BoNT/A-treated groups had decreased body weight, although the differences were not significant compared to the negative control groups. This data showed that the BoNT/A-treated mice experienced a weight loss of approximately 4% compared to the negative control groups. Overall, weight loss in the BoNT/A-treated groups was found to be the same across mice originating from the three different sources.

### Clinical pathology analysis

For clinical pathology evaluations, we conducted hematology and serum biochemistry analyses. In hematological evaluation, we analyzed a total of 10 parameters including RBC levels and compared them between the negative control groups and the BoNT/A-treated groups (Table 2). The MCV and MCH levels in the BoNT/A-treated groups were significantly higher than those in the negative control groups in mice from all three sources. The RBC, HGB, and HCT levels in the BoNT/A-treated groups were significantly lower than those in the negative control groups in mcie from all three sources. The MCHC, PLT, and WBC levels in the BoNT/A-treated groups were significantly different from those in the negative control groups in A:ICR and B:ICR mice. In terms of serum biochemistry, a total of 20 serum parameters were measured including AST and compared between the negative control groups and BoNT/A-treated groups (Table 3). The Na, K, CRE, ALP, HDL, and PL levels in the BoNT/A-treated groups were significantly lower than those in the negative control groups in mice from all three sources. The LDL level in the BoNT/A-treated groups was significantly higher than that in negative control groups in mice from all three sources. In addition, there were significant changes in several parameters with the patterns differing based on the source of the mice.

**Table 2:** Hematological analysis of Korl:ICR, A:ICR and B:ICR with BoNT/A muscle treatment

Parameters/Groups	Con-Korl:ICR	Treated-Korl:ICR	Con-A:ICR	Treated-A:ICR	Con-B:ICR	Treated-B:ICR
No. of animals	10	10	10	10	10	10
RBC (10^6^/ul)	10.55 ± 0.44	9.10 ± 0.27^**^	10.50 ± 0.25	8.61 ± 0.58^**^	10.72 ± 0.24	8.72 ± 0.25^**^
HGB (g/dl)	15.67 ± 0.71	14.67 ± 0.58^**^	15.58 ± 0.33	13.98 ± 0.75^**^	15.79 ± 0.44	14.26 ± 0.36^**^
HCT (%)	50.93 ± 1.77	47.94 ± 1.76^**^	49.21 ± 1.04	46.15 ± 2.61^**^	50.55 ± 1.13	46.44 ± 1.07^**^
MCV (fL)	48.32 ± 0.92	52.70 ± 1.47^**^	46.88 ± 0.40	53.61 ± 1.36^**^	47.16 ± 0.37	53.30 ± 1.40^**^
MCH (pg)	14.86 ± 0.20	16.14 ± 0.66^**^	14.84 ± 0.16	16.26 ± 0.58^**^	14.72 ± 0.14	16.28 ± 0.37^**^
MCHC (g/dl)	30.79 ± 0.79	30.64 ± 0.68	31.64 ± 0.35	30.31 ± 0.45^**^	31.25 ± 0.38	30.69 ± 0.41^**^
PLT (10^3^/ul)	1117.70 ± 134.98	1276.30 ± 184.56	1065.00 ± 200.56	1505.10 ± 179.39^**^	1107.60 ± 122.23	1275.50 ± 193.77^**^
WBC (10^3^/ul)	2.23 ± 0.85	2.58 ± 0.80	2.62 ± 0.77	4.98 ± 1.28^**^	1.48 ± 0.28	3.38 ± 1.11^**^
WBC Diffrential count						
Neutrophil (10^3^/ul)	0.33 ± 0.09	0.43 ± 0.14	0.37 ± 0.08	0.74 ± 0.51	0.25 ± 0.05	0.51 ± 0.13
Lymphocyte (10^3^/ul)	1.57 ± 0.48	1.94 ± 0.64	2.11 ± 0.65	3.95 ± 1.27^**^	1.15 ± 0.16	2.59 ± 0.87
Monocyte (10^3^/ul)	0.03 ± 0.01	0.05 ± 0.02	0.04 ± 0.01	0.08 ± 0.02^**^	0.03 ± 0.01	0.08 ± 0.02
Eosinophil (10^3^/ul)	0.05 ± 0.02	0.12 ± 0.06	0.07 ± 0.02	0.13 ± 0.09	0.06 ± 0.01	0.14 ± 0.18
Basophil (10^3^/ul)	0.01 ± 0.01	0.02 ± 0.01	0.01 ± 0.01	0.02 ± 0.01^**^	0.01 ± 0.01	0.02 ± 0.01
Neutrophil (%)	15.98 ± 4.76	17.09 ± 4.87	14.42 ± 1.43	15.19 ± 9.47	16.89 ± 2.74	15.87 ± 4.26^**^
Lymphocyte (%)	72.13 ± 10.14	74.57 ± 4.76	79.99 ± 2.02	78.71 ± 9.55	77.60 ± 11.75	76.14 ± 4.41^**^
Monocyte (%)	1.40 ± 0.70	2.05 ± 0.50	1.48 ± 0.24	1.58 ± 0.43	1.80 ± 0.88	2.32 ± 0.31^**^
Eosinophil (%)	2.31 ± 1.46	4.40 ± 1.86	2.68 ± 0.45	2.94 ± 2.32	4.02 ± 1.66	3.65 ± 3.10
Basophil (%)	0.55 ± 0.28	0.58 ± 0.29	0.47 ± 0.22	0.48 ± 0.18	0.79 ± 0.36	0.60 ± 0.25
Reti (10^3^/ul)	339.60 ± 30.29	318.06 ± 148.94	341.60 ± 25.56	331.18 ± 58.50	325.71 ± 28.23	355.47 ± 53.07
Reti (%)	3.23 ± 0.37	3.51 ± 1.65	3.26 ± 0.27	3.88 ± 0.87	3.04 ± 0.20	3.84 ± 0.55^**^

The data shown represent the mean ± SD (*n* = 10/group)

*Significantly different from the negative control group (*p < 0.05, **p < 0.01)

**Table 3:** Serum biochemistry analysis of Korl:ICR, A:ICR and B:ICR with BoNT/A muscle treatment

Parameters/Groups	Con-Korl:ICR	Treated-Korl:ICR	Con-A:ICR	Treated-A:ICR	Con-B:ICR	Treated-B:ICR
No. of animals	10	10	10	10	10	10
Na (mmol/L)	159.19 ± 0.79	152.95 ± 1.28^**^	156.24 ± 1.06	150.09 ± 2.05^**^	157.43 ± 1.2	153.16 ± 1.96^**^
K (mmol/L)	7.00 ± 0.05	5.65 ± 0.71^**^	6.78 ± 0.04	6.91 ± 0.55	6.90 ± 0.08	6.74 ± 0.83
CL (mmol/L)	117.81 ± 0.46	115.75 ± 3.07	115.77 ± 0.84	107.91 ± 2.57^**^	116.35 ± 1.06	111.12 ± 1.36^**^
TP (g/dL)	5.35 ± 0.05	5.44 ± 0.30	5.28 ± 0.04	5.19 ± 0.34	5.28 ± 0.06	5.23 ± 0.37
ALB (g/dL)	3.29 ± 0.03	3.41 ± 0.18	3.14 ± 0.19	3.19 ± 0.20	3.21 ± 0.03	3.14 ± 0.22
BUN (mg/dL)	20.70 ± 0.20	18.54 ± 3.55	20.36 ± 0.13	18.64 ± 3.60	20.43 ± 0.23	20.18 ± 1.96
CRE (mg/dL)	0.38 ± 0.04	0.30 ± 0.07^**^	0.39 ± 0.03	0.33 ± 0.05^**^	0.40 ± 0.00	0.30 ± 0.05^**^
GLU (mg/dL)	164.30 ± 0.95	152.80 ± 22.24	161.10 ± 1.29	210.50 ± 23.90^**^	161.60 ± 1.96	159.90 ± 13.86
TBIL (mg/dL)	0.01 ± 0.00	0.05 ± 0.05	0.01 ± 0.00	0.04 ± 0.05^**^	0.01 ± 0.00	0.05 ± 0.05
Ca (mg/dL)	10.12 ± 0.06	9.96 ± 0.41	9.98 ± 0.09	9.95 ± 0.41	10.02 ± 0.12	10.07 ± 0.45
PHOS (mg/dL)	8.62 ± 0.04	9.38 ± 1.10	8.47 ± 0.07	10.12 ± 1.06^**^	8.50 ± 0.08	9.11 ± 0.58^**^
TCHOL (mg/dL)	133.10 ± 0.74	108.10 ± 17.15^**^	130.60 ± 1.17	122.40 ± 13.79	131.20 ± 1.32	137.70 ± 28.72
TG (mg/dL)	127.80 ± 1.48	43.80 ± 19.56^**^	132.00 ± 1.41	104.40 ± 31.73	129.80 ± 1.32	108.60 ± 29.96
AST (U/L)	144.60 ± 0.70	140.40 ± 119.47	141.80 ± 1.40	86.50 ± 62.67	142.80 ± 1.03	70.40 ± 38.61^**^
ALT (U/L)	49.40 ± 0.52	36.40 ± 8.37^**^	48.50 ± 0.53	41.60 ± 19.59	48.60 ± 0.52	35.80 ± 10.96^**^
ALP (U/L)	501.10 ± 2.33	384.10 ± 87.26^**^	492.80 ± 4.08	370.10 ± 95.19^**^	494.80 ± 4.26	326.00 ± 84.85^**^
HDL (mg/dL)	120.50 ± 0.97	95.70 ± 14.01^**^	117.20 ± 1.23	103.90 ± 11.75^**^	118.80 ± 1.14	119.80 ± 24.47
LDL (mg/dL)	11.50 ± 0.18	19.35 ± 4.30^**^	11.46 ± 0.46	16.83 ± 4.38^**^	11.56 ± 0.20	16.81 ± 8.31
PL (mg/dL)	251.20 ± 3.88	189.80 ± 26.86^**^	255.00 ± 5.03	228.20 ± 21.76^**^	252.90 ± 3.87	256.20 ± 41.74
hsCRP (mg/dL)	0.01 ± 0.00	0.01 ± 0.00	0.01 ± 0.01	0.01 ± 0.00	0.01 ± 0.01	0.01 ± 0.01

The data shown represent the mean ± SD (*n* = 10/group)

*Significantly different from the negative control group (*p < 0.05, **p < 0.01). The data represent the mean ± SD

### Gene expression patterns

**Fig. 2:**
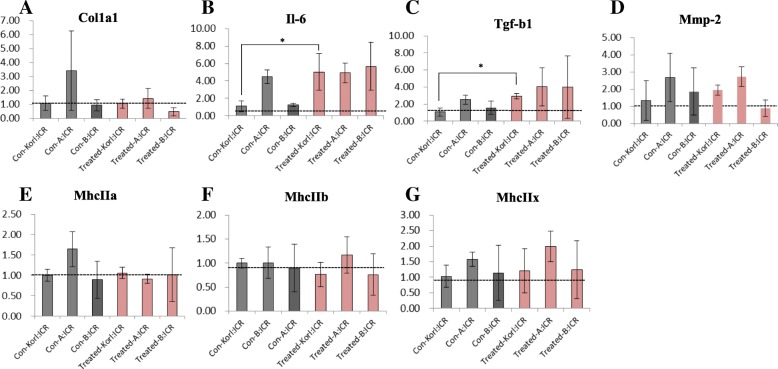
Gene expression patterns of muscle-related target genes following BoNT/A muscle injection in ICR mice from three different sources. **a**–**g** qRT-PCR comparing negative control groups and BoNT/A treated groups. Expression levels of collagen type 1 (*Col1a1*), interleukin 6 (*Il-6*), transforming growth factor beta 1 (*Tgf-b1*), matrix metallopeptidase (*Mmp-2*), myosin heavy chain IIa (*MhcIIa*), myosin heavy chain IIb (*MhcIIb*), and myosin heavy chain IIx (*MhcIIx*) were analyzed. *Significantly different compared to the negative control group (*p < 0.05). The data represent the mean ± SD of three replicates

The expression patterns of seven muscle-related genes were analyzed in the BoNT/A-treated and the negative control groups (Fig. 2). Collagen type 1 (*Col1a1*), interleukin 6 (*Il-6*), transforming growth factor beta 1(*Tgf-b1*), matrix metallopeptidase (*Mmp-2*), myosin heavy chain IIa (*MhcIIa*), myosin heavy chain IIb (*MhcIIb*), myosin heavy chain IIx (*MhcIIx*) were the target genes analyzed. The gene expression levels of *Il-6* and *Tgf-b1* showed significant upregulation in the BoNT/A-treated group compared to the negative control group in Korl:ICR mice. There was no significant difference in expression of other target genes between the groups.

### Histopathology analysis of muscle

**Fig. 3:**
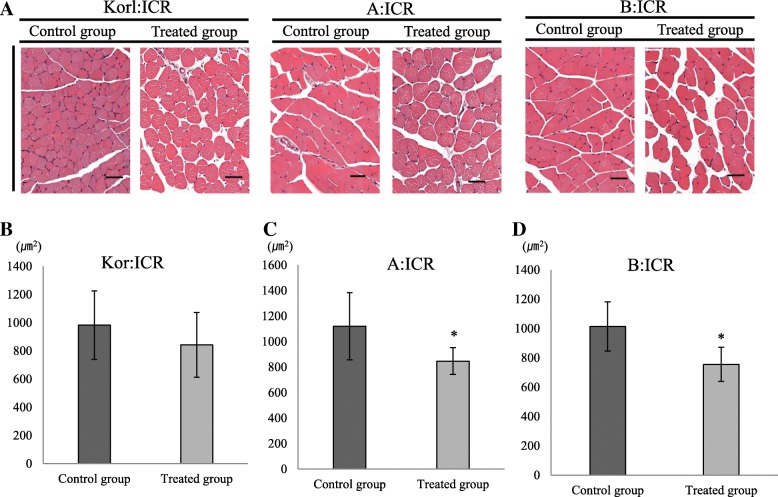
Histopathology analysis following BoNT/A muscle injection in ICR mice from three different sources. **a** H&E staining of mice muscle. Scale bar = 50 μm. **b**–**d** Muscle size was compared between the negative control and BoNT/A treated groups. *Significantly different compared to the negative control group (*p < 0.05). The data represent the mean ± SD of three replicates

For histology analysis, we performed H&E staining of mouse muscle samples after necropsy (Fig. 3). Compared with negative control groups, the change of muscle fibers was detected more commonly in the BoNT/A-treated groups. These changes, decreased muscle fibers, were obvious, while other findings including inflammation were not. Quantitative histology analysis of muscle fiber change was done using the image analysis software ImageJ. Decreased muscle fiber was confirmed in all mice in the BoNT/A-treated groups compared to those in the negative control groups. The BoNT/A-treated groups showed obvious muscle fiber change, with A:ICR and B:ICR mice showing especially significant decreased muscle fibers (Fig. 3). Overall, the above results indicated that various responses to BoNT/A muscle injection were similar in ICR mice originating from three different sources.

## Discussion

BoNT/A is used in a range of areas for various applications. For therapeutic purposes, BoNT/A’s ability to induce muscle paralysis has been clinically applied in the treatment of muscular overactivity conditions such as hyperhidrosis, chronic anal fissure, and spastic muscle diseases. In recent years, BoNT/A has been more widely used as an agent in plastic surgery for improvement of external appearance than for disease treatment. It has been applied to many muscles for cosmetic purposes, including the muscles around the jaw and calf muscles. Studies using BoNT/A have largely involved safety assessments via potency tests in laboratory animals. In addition, studies addressing mechanism and phenotype of muscle change have been reported, including those evaluating dose-dependent responses to BoNT/A muscle injection [23].

Research using laboratory animals with rodent has long been conducted on basic research and treatment of human diseases and on the pathogenesis. Using experimental animal models, various mechanisms including toxicity assessment and efficacy of disease had been used. ICR mice, an outbred rodent model, have been previously used for the study of BoNT/A [24]. In previous rodent experiments, muscle change through the administration of BoNT/A muscle was found at various concentrations [23], but no comparative study has been conducted with ICR mice originating from different sources. The present study investigated the response to BoNT/A injection into muscle using mice from three different sources, Korl:ICR, A:ICR, and B:ICR.

We assessed muscle response through histopathology evaluation after BoNT/A administration, as well as via various physiological assessments including weight measurement, and hematology and serum biochemistry analyses. To the best of our knowledge, such a range of data has not previously been reported in the context of BoNT/A administration to laboratory animals. Weight loss following BoNT/A administration was observed in all treated groups (Fig. 1). Although no behavioral tests were performed, a limitation in movement due to decreased muscle fiber caused by BoNT/A was apparent. Weight change may thus have occurred in the BoNT/A-treated groups due to reduced feed intake consequent to reduced mobility. Results of clinical pathology assessment confirmed statistically significant changes in several hematology and serum biochemistry parameters in the BoNT/A-treated groups (Tables 2 and 3). In GLU data, significant up-regulation was observed in A:ICR (Table 3). In the case of TCHOL and TG, significant down-regulation was observed in Korl: ICR (Table 3). This is thought to be a specific expression according to each mice source. These results suggest that changes in serum biochemical parameters due to the administration of BoNT/A muscle may have specificity in mice from different sources. Therefore, in the serum biochemical evaluation of BoNT/A mice, some parameters may need to consider the specificity of the each mice source. These changes in the parameter values are considered be due to the administration of BoNT/A muscle injection. However, many of the changed clinical pathologic parameter values were found to be within the normal range of reference to the previous studies [25–27]. Given that this is the first report of clinical pathology data after intramuscular administration of BoNT/A in ICR mice, our findings are meaningful and justify further experiments. We evaluated gene expression patterns of *Col1a1*, *Il-6*, *Tgf-b1*, *Mmp-2*, *MhcIIa*, *MhcIIb*, and *MhcIIx* as target markers. A previous study evaluated the expression patterns of related genes after high dose BoNT/A (6.0 U/kg) muscle injection, and found an increase in levels of *IL-6*, an inflammation-related marker, and in those of *Tgf-b1* [23]. Our results confirmed a significant increase in expression levels of *Il-6* and *Tgf-b1* in Korl: ICR. On the other hand, there was no significant difference in the expression patterns of the evaluated genes in mice from the other two sources. Based on these results, inflammation was expected to have occurred in the Korl:ICR mice, but no such findings were apparent in clinical or histopathology data. This discrepancy may have been due to the lower dose (1.0 U/kg) of BoNT/A we used, compared to that in the previous study [23]. Sprague Dawley rats (SD rats) were used for BoNT/A administration in the previous study and the results were analyzed after 21 days. The difference in the expression patterns of the target gene compared to the previous study is considerate to attribute to differences in used animal species, dose level, and duration of experiment.

In this experiment, the histopathological evaluation showed that the decreased muscle fiber resulting from the administration of the muscles of BoNT/A was generally induced (Fig. 3). Quantitative assessment of muscle change also confirmed the above findings (Fig. 3). As mentioned earlier, no inflammatory findings were accompanied.

## Conclusions

In summary, we evaluated physiological, clinical pathological, and histopathological responses to injection of BoNT/A into calf muscles of ICR mice originating from three different sources. The three different groups of mice, Korl:ICR, A:ICR, and B:ICR showed similar responses to BoNT/A injection and consequent decreased muscle fiber liesions. Our results suggest that the Korl:ICR mice are as widely applicable in muscle-related studies using BoNT/A, as other currently commercially available ICR mice.

## Data Availability

Data generated or analyzed during this study are included in this published article. The datasets generated and/or analyzed during the current study are also available from the corresponding author on a reasonable request.

## Abbreviations

A:ICR: USA source
ALB: Albumin
ALP: Alkaline phosphatase
ALT: Alanine aminotransferase
AST: Aspartate aminotransferase
B:ICR: Japan source
BoNT/A: Botulinum-toxin A
BUN: Blood urea nitrogen
Ca: Calcium
CHO: Total cholesterol
Cl: Chloride
*Col1a1*: Collagen type 1
CRE: Creatinine
GLU: Glucose
H&E: Hematoxylin and eosin
HCT: Hematocrit
HDL: High density lipoprotein
HGB: Hemoglobin
hsCRP: High-sensitive C-reactive protein
*Il-6*: Interleukin 6
IP: 'Inorganic phosphorus
K: Potassium
Korl:ICR: Korea FDA source
LDL: Low density lipoprotein
MCH: Mean corpuscular hemoglobin
MCHC: Mean corpuscular hemoglobin concentration
MCV: Mean corpuscular volume
*MhcIIa*: Myosin heavy chain IIa
*MhcIIb*: Myosin heavy chain IIb
*MhcIIx*: Myosin heavy chain IIx
*Mmp-2*: Matrix metallopeptidase
Na: Sodium
PL: Phospholipid
PLT: Platelet
RBC: Red blood cell
RET: Reticulocyte
SPF: Specific pathogen-free
TBIL: Total bilirubin
TG: Triglyceride
*Tgf-b1*: Transforming growth factor beta 1
TP: Total protein
WBC: White blood cell
